# Relationship between Salivary Alkaline Phosphatase Enzyme Activity and The Concentrations of Salivary Calcium and Phosphate Ions

**DOI:** 10.22074/cellj.2015.523

**Published:** 2015-04-08

**Authors:** Mina Jazaeri, Hosein Malekzadeh, Hamidreza Abdolsamadi, Loghman Rezaei-Soufi, Mohammad Samami

**Affiliations:** 1 Department of Oral Medicine, School of Dentistry, Hamadan University of Medical Sciences, Hamadan, Iran; 2 Department of Oral Medicine, School of Dentistry, Jundi Shapour University of Medical Sciences, Ahvaz, Iran; 3 Department of Oral Medicine and Dental Research Centre, School of Dentistry, Hamadan University of Medical Sciences, Hamadan, Iran; 4 Department of Operative Dentistry and Dental Research Centre, School of Dentistry, Hamadan University of Medical Sciences, Hamadan, Iran

**Keywords:** Alkaline Phosphatase, Saliva, Calcium, Phosphate

## Abstract

Although salivary alkaline phosphatase (ALP) can balance deand remineralization processes of enamel, there is no evidence regarding its effects on the concentrations of calcium and phosphate in saliva. The present study aims to determine the relationship between salivary ALP activity and the concentrations of calcium and phosphate in saliva. In this cross-sectional study, we evaluated salivary markers in 120 males, ages 19 to 44 years. All participants provided 5 mL of unstimulated whole saliva and the level of enzyme activity as well as calcium and phosphate concentrations were measured using a colorimetric method. Data were gathered and analyzed by statistical package for social sciences (SPSS) 13.00 using Pearson correlation test. A p value of <0.05 was considered statistically significant. The mean age of participants in the present study was 32.95 ± 8.09 years. The mean pH of saliva was 6.65 ± 0.62. Salivary parameters included average ALP activity (5.04 ± 1.866 U/dL), calcium (4.77 ± 0.877 mg/dL) and phosphate (10.38 ± 2.301 mg/dL). Pearson correlation test showed no significant relationship between ALP activity and calcium and phosphate concentrations in saliva (p>0.05). According to the results of the present study, there was no significant relation between salivary ALP activity and calcium and phosphate concentrations in saliva. However, further research is highly recommended.

Recently, interest in oral fluid ( saliva ) as a diagnostic medium or special marker for systemic or oral conditions has increased exponentially (1). Salivary components such as enzymes, immunoglobulins, inorganic materials and ions have different effects on mouth homeostasis and any change in their balance may lead to oral diseases including dental caries or periodontal problems (2,3). 

Alkaline phosphatase ( ALP ) is a nonspecific phosphomonoesterase that functions through a phophosery l intermediate to produce free inorganic phosphate (4). ALP has different isoenzymes produced by different cell types such as polymorphonuclear leukocytes, osteoblasts, macrophages and fibroblast within alveolar bone and/or salivary glands (5). It has been shown in different studies that higher ALP activity is related to periodontal disease and dental caries (6,7) and it seems that the function of this protein is relatively dependent on salivary pH and buffering capacity (8). Dental caries, as a common chronic infectious disease of the oral cavity, is a complex, multi-factorial phenomenon involved with saliva characteristics. Different biochemical characteristics of saliva, such as buffering capacity and inorganic components, may affect the development of dental caries (9,10). The normal structure of enamel consists of hydroxyapatite which contains a high amount of calcium and phosphate, thus saliva may be effective on enamel maturation and remineralization (11,12) with respect to its calcium and phosphate content (13). However, spontaneous precipitation of these ions from saliva to tooth structure cannot occur (10). 

According to Vahedi et al. (14), although buffering ALP is not related to the severity of dental caries, the ion activity product for hydroxyapatite ( IPHA ) index which indicates the amount of salivary calcium and phosphate levels is related to dental caries prevalence. Shahrabi et al. (12) in their study have found no relationship between salivary ALP and dental caries in children. The results of a study performed by Kumar and Sharma (15) showed that ALP could be a useful marker for monitoring periodontal diseases. 

The previous studies established a relationship between salivary ALP activity (15) and dental caries yet have not determined a completely understood mechanism of action. With regards to the possible effects of salivary calcium and phosphate concentrations on teeth decay (12), it is important to evaluate the relation between ALP activity and salivary calcium/phosphate concentrations. To the best of our knowledge, no clinical study has been performed to date that evaluated the relationship between salivary ALP and the amount of salivary calcium and phosphate levels. The aim of the present study was to determine the relationship between salivary ALP activity and the concentrations of calcium and phosphate in saliva. 

This cross-sectional study enrolled 120 healthy men aged 19 to 44 years who presented for routine dental care to the Oral Medicine Department of Hamedan School of dentistry. Those who had positive histories of illnesses or treatments which could cause alterations in salivary rate and composition such as diabetes, rickets, deformans osteitis, periodontal disease, history of radiotherapy or chemotherapy, dehydration, antibiotic use two weeks prior to the study, and mouth breathers, were excluded (6,16). The only inclusion criterion was regular teeth brushing at least once in 24 hours (14). Informed consent was obtained from each individual prior to collection of saliva and the oral examination. The present study was approved by the Ethical Committee of the Vice Chancellor for Research and Development at Hamadan University of Medical Sciences. 

To reduce the possibility of any circadian effect, we performed saliva collection between 9 and 11 am. A total of 5 mL of unstimulated saliva were collected in centrifuge tubes 2 hours after subjects were instructed to not eat, drink or brush their teeth, according to the Navazesh method (17). Collected samples were stored in a dish that contained ice cubes and delivered to the Biochemistry Laboratory within two hours. 

The colorimetric method was used to measure calcium concentrations with a Pars Azmoon diagnostic kit ( Pars Azmoon Co., Karaj, Iran ). In this method calcium bonds to the cresolphthalein complex and produces a purple solution in which the color intensity is proportional to the calcium concentration. To examine the color intensity, the light absorption of the solution was measured by spectrophotometer ( Jenway, Staffordshire, UK ) at a 570 nm wavelength. The calcium concentration was calculated in mg/dL (18). In a similar manner to calcium, we assessed the phosphate concentration. However, two different solutions that contained ammonium molybdate and sulfuric acid were used to produce the color solution. Light absorption of the solutions was measured by a spectrophotometer at a 340 nm wavelength. The phosphate concentration was calculated as mg/dL (19). 

The level of ALP activity ( U/dL ) was measured by the Kinetic method. In this method ALP converts 4-nitrophenyl phosphate into 4-nitrophenol and produces a yellow solution. The light absorption which matches the activity of ALP was assessed by a spectrophotometer at the 405 nm wavelength (19). 

Shortly after arriving at the laboratory delivering, 0.5 ml of each sample was used to measure the pH within 30 seconds after the placement of a pH-sensitive electrode ( Hanna Instruments^®^, Inc., Michigan, USA ). Those whose salivary pH levels were in the normal range of 6.5 to 7.5 (8) were included in the study. 

We used the statistical package for social sciences ( SPSS ) version 13.0 software for data analysis. 

The Kolmogorov-Smirnov test was used to assess the normal distribution of data. The relationships between the levels of salivary ALP and calcium, as well as phosphate were compared by the Pearson correlation test. A p value of <0.05 was considered statistically significant for all analyses. 

The mean age of the participants was 32.95 ± 8.09 years. The mean pH of saliva was 6.65 ± 0.62. Salivary parameters that included average ALP activity and calcium and phosphate concentrations are shown in table 1. According to the Kolmogorov-Smirnov test, the data had a normal distribution (p>0.05). Correlation coefficiency for the relationship between ALP activity and salivary calcium was 0.038; for the relationship between ALP activity and phosphate concentration, it was 0.048. Pearson correlation test showed no significant relation between salivary ALP activity and the concentrations of calcium and phosphate in saliva (p>0.05). Figures 1 and 2 illustrate the distribution of calcium and phosphate concentrations based on ALP activity level.

Numerous studies have evaluated the effects of ALP on oral diseases (7,12,19). However, in terms of the current research, no study evaluated the possible relationship between salivary ALP activity and the concentrations of calcium and phosphate. 

The current study included participants between the ages of 19-44 years in order to avoid possible bias in ALP activity with regards to age-related bone development or destruction. As there are numerous diseases that may alter ALP activity (16), we excluded any patients from this study who had systemic diseases. Based on the possible effects of salivary pH on the function of ALP, only those who had salivary pH (8) levels in the normal range of 6.5 to 7.5 were enrolled. 

The mean concentration of salivary calcium ( mg/dL ) in our participants ( 4.77 mg/dL ) was higher than the results achieved by Shahrabi et al. 

(12) ( 3.50 mg/dL ) and Sharma et al. (20) ( 2.2 mg/ dL ). However, the current study’s result was similar to that achieved by Vahedi et al. (14). Of note the study by Shahrabi et al. (12) was conducted on children 3-5 years of age. This significant difference between the ages of the participants of two studies might be the possible cause for the difference in results. 

The mean salivary phosphate of the present study was 10.38 mg/dL. Similar to our finding Shahrabi et al. (12) reported a concentration of salivary phosphate that ranged from 10 to 11 mg/dL. In contrast, Sharma et al. (20) reported that the mean concentration of salivary phosphate was 4.30 mg/dL. 

The mean salivary ALP activity in the current study was 5.04. Shahrabi et al. (12) reported a mean ALP activity of 8.51 and Dabra and Singh (21) reported the mean ALP activity as 2.48. Gandhy and Damle (6) reported that higher levels of ALP were related to rampant caries. In contrast, Vahedi et al. (14) and Afshar et al. (22) showed that the level of ALP was unrelated to the severity of dental caries. 

Based on the current finding the level of salivary ALP activity was not related to the concentrations of salivary calcium and phosphate. It seemed the other controlling mechanism was responsible for maintaining calcium and phosphate in a normal range. However further studies conducted on a wider population with regards to sex, age and race are strongly recommended. Based on the results of the present study, the relationship between salivary ALP activity and calcium and phosphate concentrations is not significant. 

**Table 1:** The mean and standard deviation (SD) of salivary alkaline phosphatase (ALP) activity (unit/dL) and calcium/phosphate concentrations (mg/dL)


	Mean ± SD	Minimum	Maximum

**ALP**	5.04 ± 1.866	2.1	11.9
**Calcium**	4.77 ± 0.877	3.4	7.8
**Phosphate**	10.38 ± 2.301	4.1	15.3


P value of the Kolmogorov-Smirnov test.

**Fig.1:**
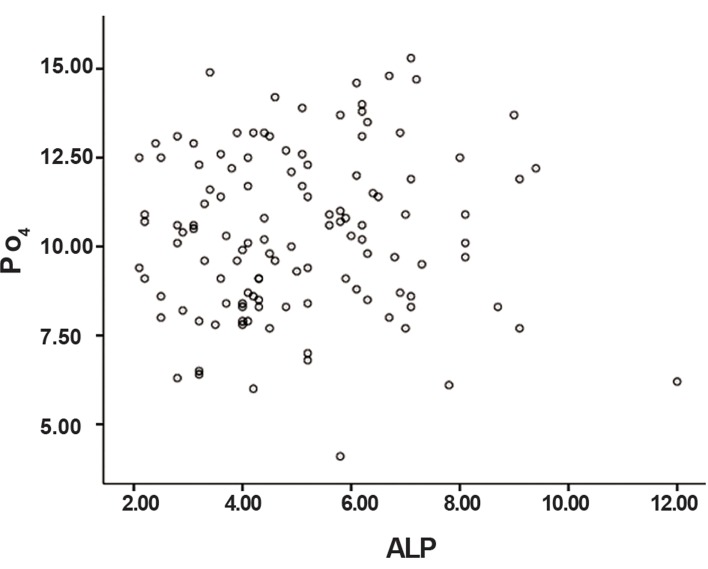
Scattered plot of the association between phosphate (PO_4_) concentrations based on alkaline phosphatase (ALP) activity.

**Fig.2:**
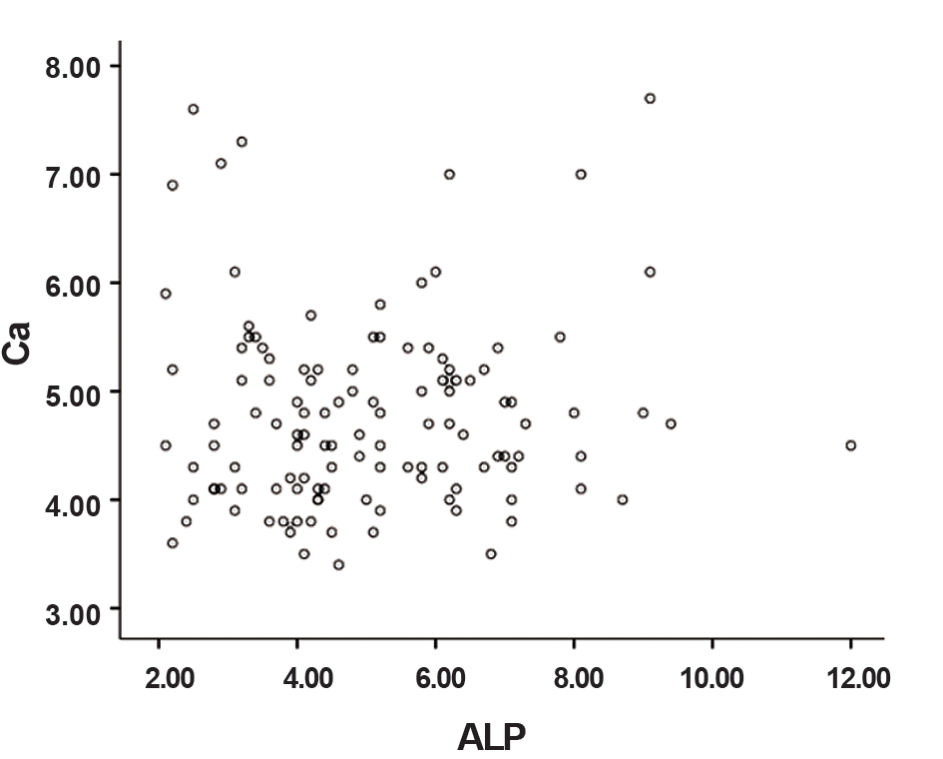
Scattered plot of the association between calcium (Ca) concentrations based on alkaline phosphatase (ALP) activity.
